# Dataset for the content of bioactive components and phytonutrients of (*Ocimum basilicum* and *Brassica rapa*) microgreens

**DOI:** 10.1016/j.dib.2021.107737

**Published:** 2021-12-20

**Authors:** Ali J. Othman, Ekaterina S. Vodorezova, Majd Mardini, Muhammad B. Hanana

**Affiliations:** a Department of Commodity Research and Commodity Expertise, Plekhanov Russian University of Economics, Moscow, Russian Federation; b Center of Molecular Biotechnology, Russian State Agrarian University - Moscow Timiryazev Agricultural Academy, Moscow, Russian Federation; c Department of Field Crops, Faculty of Agriculture, University of Damascus, Syria

**Keywords:** Microgreens, Organic acids, Total phenolic content, Phytonutrients, Pigments, Phytotron

## Abstract

The data provided in this article were obtained from fresh and dry samples of green Basilic (*Ocimum Basillicum L.*), red Basilic (*Ocimum basilicum 'purpurascens'*), green Mizuna (Brassica rapa var. niposinica), and red Mizuna (*Brassica rapa* var. pipposinica) microgreens grown in climatic chamber (phytotron) on jute mats for 15 days. Phytonutrients contents including chlorophylls, pheophytins, carotenoids pigments, total antioxidant capacity, total phenolic content, ascorbic acid, as well as organic acids contents varied between all cultivars. Spectrometry, electrophoresis, coulometric, and liquid chromatography-electrospray ionization quadrupole time-of-flight mass spectrometry (LC-ESI-QTOF/MS) were the principal employed methods. Data of antioxidants and phytonutrients contents contribute to the understanding of the benefits of microgreens as a newly emerging product. Data of pigments content compares the difference of accumulation of chlorophylls, pheophytins, and carotenoids between red and green cultivars of the studied microgreens, and the variability of their concentrations along with the contents of organic acids provide insights to plants physiology during the differentiation phase.

## Specifications Table

**Table:** 

Subject	Food Science: Food Chemistry
Specific subject area	Biotechnology, Food Chemistry, Plant Science
Type of data	Table
How the data were acquired	Shimadzu Prominence 20 HPLC UFLC system (Shimadzu, Japan) including Software Shimadzu LC Solutions UPLC-PDA-Q/TOF-MS (Waters Corporation, USA) UPLC-PDA-FL (Waters Corporation, USA) Mass detector G2 Qtof mass spectrometer (Waters Corporation, Manchester, UK) Coulometric analyzer EXPERT-006 (Econix-Expert, Russian Federation) Spectrophotometer (Shimadzu UV 2401pc, Japan) Capillary electrophoresis Capel m-105 (Lumex, Russian Federation) Moisture analyzer MX-50 (A & D, Japan) Vacuum oven (Gallenkamp, UK) MassLynx Version 4.1 software and ChromaLynx application manager
Data format	Raw and analyzed data
Description of data collection	The data on organic acids content were obtained using Shimadzu Prominence 20 HPLC UFLC System according to Sánchez [1] The data on total phenolic content were obtained by the colorimetric method of Folin–Ciocalteu using gallic acid as a standard [2]. The data on ascorbic acid content was obtained by capillary zone electrophoresis method according to Komarova and Kamentsev [3]. The data on total antioxidants capacity was obtained by coulometric analysis method using electrogenerated bromine radicals as described by Lapin A, Timofeev, and Zelenkov [4]. Ultra-performance liquid chromatography photodiode detector-quadrupole/time-of-flight mass spectrometry (UPLC-PDA-Q/TOF-MS) for identification and Ultra-performance liquid chromatography–photodiode array–fluorescence (UPLC-PDA-FL) (Waters Corporation, USA) for quantification of Carotenoids, Chlorophylls (a, b) and Phenophytin (a, b) as described by Wojdylo [5].
Data source location	Plekhanov Russian University of Economics, Stremyanny avenue, 36, Moscow, 115093, Russia Institution: Plekhanov Russian University of economics Russian State Agrarian University - Moscow Timiryazev Agricultural Academy, Timiryazevskaya street, 49, Moscow, 127422, Russia
Data accessibility	• With the article

## Value of the Data

• The contribution of Brassicaceae and Lamiaceae vegetables to health improvement may be attributed to their antioxidant capacity since they generally accumulate high levels of antioxidant phytochemicals such as ascorbic acid, carotenoids, and phenolic compounds.
• Data on organic acids, total carotenoids, chlorophylls (a and b), and pheophytins (a and b) contents provide an understanding of the plant physiology at the phase of microgreens.
• These data are useful for microgreens growers by providing additional information regarding the nutritional profile of the studied cultivars. They are as well useful to researchers interested in microgreens cultivation under closed environment systems (phytotrons).

## 1 Data Description

This dataset contains analyzed data obtained by high-performance liquid chromatography (HPLC), Ultra-performance liquid chromatography photodiode detector-quadrupole/time-of-flight mass spectrometry (UPLC-PDA-Q/TOF-MS), Ultra-performance liquid chromatography–photodiode array–fluorescence (UPLC-PDA-FL), coulometric analysis, spectrophotometry, capillary zone electrophoresis, and coulometric methods from fresh and dried samples of four soilless-grown green Basil (Ocimum basilicum L.), red Basil (Ocimum basilicum 'Purpurascens'), green Mizuna (Brassica rapa L. ssp. nipposinica), and red Mizuna (Brassica rapa L. ssp. nipposinica) microgreens cultivars grown in a climatic chamber (phytotron). Dataset provided about organic acids, pigments contents, ascorbic acid, total phenolic content, dry matter, and total antioxidant capacity are displayed in Table 1.

**Table 1:** Dry matter, organic acids, chlorophyll a, chlorophyll b, pheophytin a, pheophytin b, total phenolic content, ascorbic acid, and anti-oxidant capacity of green basil (*Ocimum basilicum L.),* red basil (*Ocimum basilicum 'purpurascens'*), mizuna green (*Brassica rapa var. niposinica),* and mizuna red (*Brassica rapa var. pipposinica*) soilless grown microgreens cultivars. All data are expressed as mean ± standard error, n = 4.

	Compounds	Green Basil (*Ocimum basilicum L*.)	Red Basil (*Ocimum basilicum* 'purpurascens')	Mizuna Green (*Brassica rapa* var. niposinica)	Mizuna Red (Brassica rapa var. pipposinica)
Organic acids	Oxalic acid (g/100 g DW)	1.23 ± 0.09 d	1.57 ± 0.08 c	2.36 ± 0.18 b	2.92 ± 0.23 a
	Citric acid (g/100 g DW)	0.2 ± 0.01 bc	0.11 ± 0.01 c	0.24 ± 0.01 a	0.22 ± 0.03 b
	Malic acid (g/100 g DW)	0.64 ± 0.88 b	0.06 ± 0.04 d	0.175 ± 0.01 c	1.8 ± 0.05 a
	Quinic acid (g/100 g DW)	0.36 ± 0.06 a	0.17 ± 0.0 b	0.14 ± 0.04 c	0.17 ± 0.0 b
	Succinic acid (g/100 g DW)	0.32 ± 0.06 c	0.41 ± 0.02 b	0.51 ± 0.01 a	0.5 ± 0.10 ab
	Chlorophylls a (mg/g) FW	33.7 ± 1.79 b	31.66 ± 1.36 c	35.38 ± 1.52 a	30 ± 1.40 d
	Chlorophylls b (mg/g) FW	5.44 ± 0.33 c	4.45 ± 0.15 d	5.88 ± 0.45 b	6.01 ± 0.16 a
Pigments	Pheophytin a (mg/g) FW	13.79 ± 0.90 c	10.99 ± 0.61 d	24.45 ± 1.51 b	27.95 ± 2.63 a
	Pheophytin b (mg/g) FW	3.73 ± 0.21 c	4.02 ± 0.12 b	7.71 ± 0.17 a	7.58 ± 0.24 a
	Total Carotenoids (mg/g) FW	51.5 ± 30.15 c	53.71 ± 4.05 b	70.54 ± 3.97 a	69.23 ± 4.79 a
	Total Phenolic Content (mg GAE/g DW)	642.64 ± 18.37 b	873.15 ± 6.75 a	349.5 ± 12.50 d	474.29 ± 9.03 c
	Ascorbic Acid (mg/100 g FW)	65.68 ± 2.49 b	105.87 ± 6.54 a	32.86 ± 1.90 d	44.9 ± 4.03 c
	Anti-Oxidant Capacity (mg DE/g FW)	16.26 ± 1.08 b	18.06 ± 0.85 a	8.35 ± 0.60 d	11.6 ± 0.25 c
	Dry matter (mg/100 g FW)	6.32 ± 0.20 b	7.83 ± 0.06 a	5.42 ± 0.22 c	5.77 ± 0.11 bc

Different letters within each row indicate significant differences according to Duncan's multiple range test (*P* = 0.05).

## 2 Experimental Design, Materials and Methods

### 2.1 Plant cultivation and experimental treatments

Microgreens samples cultivation was carried out in climatic chamber (phytotron ISR 0.1) conditions. Cultivars were grown from 12th until 28th of July 2021.

Fifty seeds of each cultivar were sown separately on the growing substrates based on jute with dimensions of (17.5*47 cm) and placed in plastic pots (55*90 cm). All studied cultivars were drip-irrigated with mineral water Chernogolovka® (Aqualife, Moscow Russian Federation) of the subsequent contents in mg.L^−1^: HCO^3−^ 122, Cl^−^ 11, SO₄^2−^ 36, Mg^2+^, K+ 28, Na^+^ 22. pH: 6.7–6.9, EC: 1.9 mS.cm^−1^, and TDS: 261 mg.L^−1^. Upon the third day, germinated seeds were subjected to a photoperiod of 18 h and 23/16 °C (day/night) temperature. Photosynthetic photon flux density (PPFD) of 42 µmol m^−2^.s^−1^ with red: blue = 2:3 light-emitting diodes (LEDs). The experiment comprised of randomized complete block design (RCBD), replicated 5 times for each cultivar. Microgreens were grown for 15 days after germination. Upon harvesting, the cotyledon stems were cut using sterile scissor. Samples were exposed to dry by using a vacuum oven (Gallenkamp, UK) at 70 °C for 12 h in the vacuum of 500 mbar and then placed in tightly closed brown jars for performing further analysis.

### 2.2 Organic acids content

Then contents of organic acids (malic, oxalic, quinic, citric, and succinic acids) were performed following the method described by Sanchez [1]. Exactly five grams from each microgreen's samples were weighed and then exposed to dry by using a vacuum oven (Gallenkamp, UK) at 70 °C for 12 h in the vacuum of 500 mbar and then placed inside 50 mL tightly closed Erlenmeyer flask and then placed in −18 °C freezer for 12 h. Samples were removed and homogenized with a mortar and pestle. Then 500 mg of the macerates were placed in a 250 mL beaker and mixed with 20 mL distilled water, placed on a magnetic stirrer, and boiled for 15 min at 120 °C. Mixture left to cool down for 30 min and then vortexed using digital vortex mixer (Thermo Scientific™, Denmark). The homogenized mixture was placed in an ultrasonic water bath Bandelin SONOREX™ for 10 min at a temperature of 80°C and then centrifuged at 15000 rpm for 10 min. Purification from polyphenols and delivering the aqueous phase was performed using Sep-Ak C18 cartridge for column chromatography. Exactly 1 mL of the supernatant (aqueous phase) was filtered using a gridded millipore membrane filter of pore size 0.45 µm (Merc), 20 µL of the filtrates was inserted into a Shimadzu Prominence 20 HPLC UFLC System. Organic acids were separated on using two cartridges, ABZ+Plus SupelGuard™, particle size 5 µm, (2 cm × 4 mm L × I.D.) and SUPELCOGEL™ C610H, 6% Crosslinked HPLC Column, 9 µm particle size, (30 cm × 7.8 mm L × I.D.). Phosphoric acid 0.1% was used as eluent with a flow rate of 0.5 mL/min. Organic acids were determined in quadruples. Samples were detected at 210 nm wavelength. Results were expressed in mg/100 g dried weight.

### 2.3 Chlorophyll, carotenoids, and pheophytin

Carotenoids from microgreens leaves were determined using an acquity system, the method was previously described by Wojdylo [5]. Exactly 10 µL of the fresh sample was injected in BEH RP C18 column (2.1*100 mm, 1.7 um; waters corporation.; Ireland) at temperature 31 °C with gradient elution at a flow rate of 0.5 mL/min for 16.60 min. The mobile phase was composed of solvent A 2.0% formic acid (CH_2_O_2_) and solvent B 99.8% pure acetonitrile (CH_3_CN). The remaining parameters for LC-PDA-Qtof-ESI-MS were as follows: scanning from m/z 100 to 1200; capillary voltage of 2000 V; cone voltage of 35 V; source and desolvation temperature of 100 and 250 °C, respectively; and flow rate of the desolvation gas (nitrogen) 350 L/h.

The characterization of the single component was carried out via the retention time and the accurate molecular masses at positive ion mode, and it was set to the base peak intensity (BPI) chromatograms. Retention times (Rt) and spectra (λ) were compared with those of pure standards. Quantification was achieved by injection of solutions of known concentrations ranging from 0.05 to 0.5 mg/mL (R2 ≤ 0.9977). Carotenoids detection (lutein, zeaxanthin, and ß-carotene) were at 450, 470, and 471 nm, respectively. For chlorophylls (chlorophylls a, chlorophyll b, pheophytin a, pheophytin b) at 663, 646, 660, 631 nm, respectively. All samples were assayed in triplicate, and the results were expressed as mg per kg of fresh weight (FW).

### 2.4 Total antioxidants capacity

Anti-Oxidants capacity (AOC) for microgreens was performed using a coulometric analyzer (Econix-Expert, EXPERT-006, Russia) as described by Timofeev [4]. 5 g of microgreens cuts were thoroughly macerated and then sonicated in an ultrasonic water bath Bandelin SONOREX™ for 20 min at a temperature of 80 °C. Then 1 g of the mixture was transferred into an electrolytic cell containing buffer solution (0.2 M KBr and 0.1 M H_2_SO_4_). The initial and end values of electro-titration were set on 200 mV electrical current. Bromine anions were generated under 50 mA electrical current where all compounds with anti-Oxidants properties would react with the excessive bromine anions. The electrolysis process initiated at 40 mV. Total anti-oxidants capacity expressed as Dihydroquercentin equivalent (mg DE/g) of fresh weight.

### 2.5 Ascorbic acid content

The concentration of ascorbic acid in the studied samples was determined by the method described by Komarova with slight modifications [3,6]. A 10 g of fresh microgreens shoots were gently softened and ground then transferred to a volumetric flask and diluted to 100 mL, vortexed for 15 min under temperature 70 °C, filtered, and centrifuged under the speed of 14000 rpm for 10 min. The supernatant was removed and centrifuged again to ensure maximum purity. Purified samples were placed into capillary zone electrophoresis (Kapel 105M, Lumex, Russia) under conditions of positive voltage polarity +20 kV, 24 °C, samples injected under a pressure of 450 450 mbar.s^-1^ into silica capillary of 50 µm and total length of 50 µm. Ascorbic acid was detected at A254 nm absorbance.

### 2.6 Total phenolic content

The total phenolic content for the samples were analyzed using the modified Folin–Ciocalteu colorimetric procedure based on the formation of a light blue molybdenum-tungsten complex when mixed with gallic acid [2]. Microgreens fresh cuts were exposed to dry by using a vacuum oven (Gallenkamp, UK) at 70 °C for 12 h in the vacuum of 500 mbar and then placed in tightly closed brown jars for performing further analysis. A 50 mg of the dry sample was macerated for 5 min with 2 mL of ice-cold 95% methanol. The homogenized mixture was then centrifuged at a rotation speed of 13,000 rpm for 10 min at a temperature of 23 °C. Then, 1 mL of supernatant was mixed with 2.5 mL of 10% (w/v) Folin–Ciocalteu reagent. Subsequently, 2 mL of 20% sodium carbonate solution was added to the mixture and stirred at laboratory temperature for 3 min then incubated at 45°C for 20 min, centrifuged at a rotation speed of 13,000 rpm for 10 min. Aliquots of this sample were filtered through a 0.45 µm Whatman filter. total phenolic content were measured using a spectrophotometer (Shimadzu UV 2401pc UV-VIS, Japan) at 765 nm absorbance every 3 min. The content was standardized with gallic acid and expressed as mg gallic acid equivalents (GAE) per gram of dry mass of microgreens samples using gallic acid calibration curve (R2 = 0.978).

## Ethics Statement

None.

## CRediT authorship contribution statement

**Ali J. Othman:** Conceptualization, Visualization, Methodology, Software, Formal analysis, Writing – original draft. **Ekaterina S. Vodorezova:** . **Majd Mardini:** Methodology, Software, Formal analysis. **Muhammad B. Hanana:** Formal analysis, Writing – original draft.

## Declaration of Competing Interest

The authors declare that they have no known competing financial interests or personal relationships which have, or could be perceived to have, influenced the work reported in this article.

## Appendix Supplementary materials

Image, application 1
